# Statistical Report of the Mercy Hospital for the Year 1853

**Published:** 1854-03

**Authors:** 


*Statistical Report of the Mercy Hospital for the year* 1853.

THE whole number of patients admitted into the Mercy Hospital in Chicago, from Jan. 1st., 1853, to Dec. 31st of the same year, was . . . . 447

Males, . . . . . 361

Females, . . . . 86=447

Number whose expenses were paid chiefly by the County, . . . . 109

Number whose expenses were paid by themselves or friends, . . . . 314

Number received without pay, . . 24=447

Of the whole number, 300 were admitted into the medical wards, of whom 35 died. Of the remaining 147 surgical cases, 18 died. The total number of deaths during the year was 53; of which 26 took place within 24 hours after admission, and were either from severe injuries or disease in its last stage of progress.

The following is a list of the more important diseases, viz:

| | | | | | | |
|---|---|---|---|---|---|---|
| Phthisis Pulmonalis, | 13 | cases, | 7 | deaths, | 6 | Improved. |
| Pneumonia, | 14 | " | 5 | " | 9 | Recovered. |
| Other Diseases of Lungs, | 7 | " | 1 | " | 6 | " |
| Dysentery, | 27 | " | 7 | " | 20 | " |
| Diarrhœa and Cholera Morbus, | 20 | " | 2 | " | 18 | " |
| Intermittent and Remittent Fevers, | 46 | " | 1 | " | 45 | " |
| Typhoid Fever, | 59 | " | 4 | " | 55 | " |
| Typhus Fever, | 4 | " | 2 | " | 2 | " |
| Delirium Tremens, | 8 | " | 1 | " | 7 | " |
| Rheumatism, | 8 | " | 0 | " | 8 | " |
| Inflammation of Heart, | 3 | " | 0 | " | 3 | " |
| All other Diseases, | 91 | " | 5 | " | 86 | " |
| Total, | 300 | " | 35 | " | 265 | " |
| Surgical Cases, | 147 | " | 18 | " | 129 | " |
| Total, | 447 | " | 53 | " | 394 | " |

Medical Officers at present are as follows:

| | | |
|---|---|---|
| In the Surgical Department, | | D. Brainard, M. D.<br>W. B. Herrick, M. D.<br>J. E. McGirr, M. D. |
| Medical Department, | . . . . | N. S. Davis, M. D. |
| Interine or Resident Assistant, | . | C. C. Cornett, M. D. |

